# Chronotherapy with defective circadian clock?

**DOI:** 10.18632/aging.101430

**Published:** 2018-04-24

**Authors:** Yuan Zhang, Francis Lévi, Yunhua Chang

**Affiliations:** 1 INSERM UMR935, Modèles de Cellules Souches Malignes et Thérapeutiques, Villejuif, France; 2 Université Paris-Sud, Orsay, France; 3 Division of Biomedical Sciences and Zeeman Institute: SBIDER, Warwick Medical School, University of Warwick, CV4 7AL Coventry, UK

**Keywords:** breast cancer, circadian clock, Everolimus, chronotherapy

In mammals, the Circadian Timing System (CTS) involves a central pacemaker in the brain (hypothalamic suprachiasmatic nuclei), which coordinates a network of molecular clocks in each cell from all organs, through the generation of rhythmic physiology and hormonal secretions about 24 hours [1,2]. The molecular clock in cells is made of interwoven transcription and translation feedback loops involving 15 clock genes and proteins, which rhythmically regulate the majority of transcription and/or translation processes, with organ-specificity [1,2]. The central pacemaker further adjusts the network of peripheral clocks to the environmental light-dark and other cycles.

The circadian rhythms, with an about 24-hour cycle, could significantly modify the efficacy and toxicity mechanisms of more than 50 anticancer drugs [1]. Strikingly optimally-timed circadian delivery schedules (chronotherapy) have jointly improved both tolerance and efficacy of treatments, both at preclinical and clinical levels. In international randomized clinical trials, a fixed chronotherapy schedule improved tolerability up to 5-fold and nearly doubled efficacy as compared to constant rate, or oppositely-timed infusions in patients with metastatic colorectal cancer [1,2]. However, major findings from human and cell lines studies indicate that during breast cancer process, circadian timing system is often distorted in breast cancer patients as well as in breast tumors and breast cancer cells lines [3]. This aspect is calling for identifying chronoefficacy mechanisms of some anticancer drugs at breast cancer cells level, which could be possibly translated into chronotherapy for breast cancer.

Everolimus (EV), a selective inhibitor of mammalian target of rapamycin complex 1 (mTOR1), inhibits the synthesis of cell cycle and glycolysis proteins, resulting in both immunosuppression and anticancer effects [4]. A combination of anti-estrogens with EV is currently used to treat Estrogen-Receptor positive (ER+) breast cancers that are resistant to endocrine therapies [4]. Matched to such clinical application, we have chosen ER+ breast cancer cell line MCF-7 as a model for the determination of administration-time-dependent efficacy of EV. It is puzzling to discover that EV inhibited MCF-7 cell proliferation with an extent varied according to in vitro administration timing [5], despite that no evident circadian rhythm of the canonical clock genes expression was found in MCF-7 cells.

Yet, exogenous signals such as serum shock successfully synchronized mTOR activity and cell cycling in our study [5], as well as more than 400 mRNA expressions with an about 24-hour period [3]. We found that the 24-hour oscillation of mTOR activity might in turn entrain or trigger oscillatory expressions or phosphorylation of G1- to S-phase progression proteins, including Cyclin D1 and RB. Thus serum-shocked MCF-7 cells presented apparently rhythmic changes in both mTOR activity and cell cycle progression probabilities, that provided a rationale for possible time-dependent EV sensitivity in “molecular clock-deficient” MCF-7 cells.

Indeed, EV efficacy, as assessed with Go-G1 phase cells accumulation, varied up to four-fold as a consequence of different dosing time over the 24 hours. This striking finding provides not only some useful hints toward EV chronotherapy in human ER+ breast cancers, but also support chronotherapeutics with other anti-cancer drugs, irrespective of canonical genetic circadian clock function in cancer cells. As more and more clock gene-independent rhythms are being discovered, such as the highly conserved peroxiredoxins biochemical circadian clock or the cell autonomous 12-hour clock in mouse liver etc [6,7], clock gene dependent and independent rhythmic pathways deserve to be considered for the optimization of cancer chronotherapy through a systems approach [2].

Thus, we have shown that, in a breast cancer cell system with disrupted circadian timing system, integrating others cell rhythms in chronotherapy could also increase drug efficacy. This principle may apply to many other cancer systems and treatment types.
